# Effect of Traditional Chinese Herbal Medicine with Antiquorum Sensing Activity on *Pseudomonas aeruginosa*

**DOI:** 10.1155/2013/648257

**Published:** 2013-11-11

**Authors:** Weihua Chu, Shuxin Zhou, Yan Jiang, Wei Zhu, Xiyi Zhuang, Jiangyan Fu

**Affiliations:** ^1^Department of Microbiology, School of Life Science & Technology, China Pharmaceutical University, Nanjing 210009, China; ^2^School of International Pharmaceutical Business, China Pharmaceutical University, Nanjing 210009, China; ^3^Jiangsu Entry-Exit Inspection and Quarantine Bureau, Nanjing 210001, China

## Abstract

Traditional Chinese herbal medicines (TCHMs) were tested for their ability of antiquorum sensing. Water extracts of *Rhubarb, Fructus gardeniae, and Andrographis paniculata* show antiquorumsensing activity when using *Chromobacterium violaceum* CV12472 as reporter; the sub-MIC concentrations of these TCHMs were tested against AHL-dependent phenotypic expressions of PAO1. Results showed significant reduction in pyocyanin pigment, protease, elastase production, and biofilm formation in PAO1 without inhibiting the bacterial growth, revealing that the QSI by the extracts is not related to static or killing effects on the bacteria. The results indicate a potential modulation of bacterial cell-cell communication, *P. aeruginosa* biofilm, and virulence factors by traditional Chinese herbal medicine. This study introduces not only a new mode of action for traditional Chinese herbal medicines, but also a potential new therapeutic direction for the treatment of bacterial infections, which have QSI activity and might be important in reducing virulence and pathogenicity of pathogenic bacteria.

## 1. Introduction

The emergence of multiresistant bacteria (super bacteria) due to the misuse of antibiotic led to the search for alternative approaches other than those using antibiotics which are needed in the fight against infectious diseases. Quorum sensing (QS) or cell-to-cell communication is a cell density-dependent bacterial response mediated by hormone-like compounds called autoinducers [1]. QS-dependent regulation of gene expression controls a wide variety of prokaryotic phenotypes including biofilm formation, virulence factor expression, and motility [2, 3]. Quorum sensing inhibition (QSI) is considered as a new approach of antimicrobial chemotherapy as anti-QS compounds target genes that are essential for basic metabolism in vitro, rather than the microorganisms itself [4–6].

Traditional Chinese herbal medicines (TCHMs) are a major aspect of traditional Chinese medicine practice in China, which focuses on restoring a balance of energy, body, and spirit to maintain health rather than treating a particular disease or medical condition. TCHMs have been used for thousands of years. TCHMs are used increasingly in western countries in conjunction with or in place of allopathic medicine, as they are considered to be effective and to have few side effects. They have been used extensively to combat bacterial infections, and their biocidal or biostatic activities have been described [7]. But little is known, however, about whether their effectiveness is also due to QSI. Quorum sensing inhibitors are proposed as alternative therapeutics to overcome the problem of increasing antibiotic resistance of bacteria in infectious diseases [8, 9]. 

The object of this study was to investigate the ability of traditional Chinese herbal medicines (TCHMs) to modulate the expression of QS-dependent virulence factors in *P. aeruginosa*.

## 2. Materials and Methods

### 2.1. Collection of Traditional Chinese Herbal Medicines and Extract Preparation

Five “Qingre” herbs (“cleansing heat”) used in this study (Table 1) were bought from the store of Chinese medicine in Nanjing, China, including *Rhubarb*, *Rhizoma coptidis*, *Cortex phellodendri Chinensis*, *Fructus gardenia,* and *Andrographis paniculata*. The herbs were then ground to powders using a mechanical grinder. Powders were extracted by maceration in water. Approximately 50 g of the powdered materials were boiled in 200 mL of water for 3 × 60 min. The solvent was then removed under reduced pressure in a rotary evaporator (N-1000S, EYELA, Japan). The respective solvents were filtered through Whatman filter paper No. 1 and concentrated on a rotary vacuum evaporator. Concentrated extracts were then dried in a laminar hood and were further dried in a desiccator to obtain the crude extract. The crude extract was reconstituted in water to a concentrate of 1 g of starting herbal material per mL (1 g/mL). Filtration of the water extracts using a 0.22 *μ*m (pore size) filter into autoclaved vials ensured sterility of the samples. Extracts were tested for microbial contamination at every step of processing, by streaking to LB agar plates, to minimize the potential for introduction of exogenous anti-QS compounds. The extracts were stored at −20°C.

### 2.2. Bacterial Strains and Culture Conditions

*C. violaceum* CV12472 is a wild strain and produces QS controlled purple pigment, violacein. It produces and responds to the cognate autoinducer molecules C4 and C6 AHLs [10]. *P. aeruginosa* (PAO1) is a pathogenic strain and many traits including swarming motility are under QS regulation [11]. Unless otherwise stated, all strains were grown in Luria-Bertani (LB, 5 g yeast extract, 10 g tryptone, 5 g NaCl, 1 L water) solidified with 1.5% agar when required. Growth temperatures for *C. violaceum* and *P. aeruginosa* were 30°C and 37°C, respectively, with 150 rpm agitation in a shaking incubator, overnight.

### 2.3. Antiquorum Sensing Assay

The bacterial biomonitor strain *C. violaceum *ATCC 12427 was a kind gift from Professor Robert Mclean of the Department of Biology, Texas State University-San Marcos, USA. This strain produces a purple pigment, violacein, which is under quorum sensing control. The inhibition of violacein production by antiquorum sensing material makes this bacterium an excellent model for the isolation of antiquorum sensing substances from natural products. The agar well diffusion assay was adopted to detect anti-QS activity using the method described previously [12].

The agar well diffusion assay was employed to test the antiquorum sensing activity of the different extracts of different TCHMs. The agar well diffusion assay was performed by *C. violaceum* CV12472 for determining pigment inhibition activity by TCHMs. Luria plates were spread with 0.1 mL of appropriately diluted (~3 ×  106 CFU/mL) freshly grown cultures and wells of 8 mm diameter were made and sealed at the bottom by soft agar. Then 100 *μ*L of stock solution (~1 g/mL) were added in the well. Water was used as negative control. Plates were incubated at 30°C for 24 h to check the inhibition of the pigment production around the well.

### 2.4. Effect of TCHMs Extract on Bacterial Growth

Minimum inhibitory concentrations (MICs) for the extracts of TCHMs were determined against *P. aeruginosa* by macrobroth dilution technique as described by Aqil et al. [13]. The MIC was defined as the lowest concentration at which there is no visible growth of the test bacteria. A range of concentrations below MIC value (Sub MIC) of the extract was taken to assess their effect on the QS of *P. aeruginosa*.

Flask incubation assay was performed to evidence the non-growth-inhibitory activity of test extract. One percentage overnight culture of *P. aeruginosa* (OD_600_ adjusted to 0.4) was inoculated in 100 mL Erlenmeyer flasks containing 50 mL of LB broth supplemented with sub-MIC concentration of TCHMs extract (*Rhubarb*: 0.0625 g/mL, *Fructus gardeniae*: 0.25 g/mL, *Andrographis paniculata*: 0.125 g/mL). The flasks were incubated at 37°C with 150 rpm agitation for 24 h in a rotatory shaker. Cell density was measured in UV-visible spectrophotometer at every 1 h interval.

#### 2.4.1. Effect of TCHMs Extract on QS-Dependent Virulence Factors in *P. aeruginosa* PAO1

PAO1 was grown in LB supplemented to contain 1% extract for 24 hrs at 30°C and then centrifuged (8000 rpm, 4°C, 10 min), and the virulence factors were determined in cell-free supernatants using standard protocols.

#### 2.4.2. Protease and Elastase Assay

For protease and elastase assay, the TCHMs at the sub-MIC concentration were added in 2 mL of LB broth inoculated with 1% (20 *μ*L) of PAO1 culture (0.4 OD at 600 nm). TCHMs were omitted in control experiments. All cultures were incubated at 37°C for a minimum of 18 h. After incubation, the protease activity was determined by a skim milk plate assay [14]. In brief, 100 *μ*L of cell-free supernatant of TCHMs-treated and -untreated PAO1 was separately inoculated on LB solid medium containing 2% skim milk, and incubated at 37°C for 24 h, the zone of casein hydrolysis was detected. The elastolytic activity was determined by following the method of Ohman et al. [15] using Elastin Congo Red (ECR) (Sigma, St. Louis, USA) as the substrate. In brief, 100 *μ*L of treated and untreated PAO1 culture supernatant were added into 900 *μ*L of ECR buffer (100 mM Tris and 1 mM CaCl_2_) (pH 7.5) containing 20 mg of ECR and incubated with shaking at 37°C for 3 h. The reaction was stopped by adding 1,000 *μ*L of 0.7 M sodium phosphate buffer (pH 6.0). The tubes were placed in an ice water bath for 15 min and centrifuged to remove insoluble ECR. The absorbance of the supernatant was measured at OD_495_.

#### 2.4.3. Pyocyanin Assay

The pyocyanin assay was performed based on the absorbance of pyocyanin at 520 nm in acidic solution [16]. Cell-free supernatant of PAO1, cultured in the presence and absence of TCHMs extracts, was extracted with 12 mL of chloroform and then reextracted with 4 mL of 0.2 N HCl to give a pink to deep red solution. The absorbance of the solution was measured at OD_520_.

#### 2.4.4. Swimming Assay

The swimming motility was assessed as described by Packiavathy et al. [17]. Ten microliters of PAO1 was point-inoculated at the center of the swimming agar medium containing 1% (w/v) tryptone, 0.5% NaCl, and 0.3% agar along with the sub-MIC concentration of TCHMs. Swim agar plate without the addition of TCHMs was maintained as control. The plates were incubated at 37°C in upright position for the period of 24 h.

#### 2.4.5. Biofilm Formation Assays

PAO1 culture was inoculated in fresh LB medium in the presence or absence of plant extracts as mentioned above. Tubes were incubated for 12 h at 37°C. After incubation, planktonic cells and spent media were discarded, and adherent cells were gently rinsed twice with deionized water and allowed to air-dry before being stained. The biofilms were stained by 0.4% crystal violet solution for 5 min, after which tubes were rinsed twice with deionized water and absorbed dye was eluted with ethanol. The absorbance of dye in ethanol was measured at OD_650_ [18].

### 2.5. Statistical Analysis

All the experiments were performed in triplicate to validate reproducibility and the *P* values were calculated statistically by Student's *t* test. 

## 3. Results

### 3.1. Anti-QS Properties of Water Extracts of TCHMs

**Figure 1:**
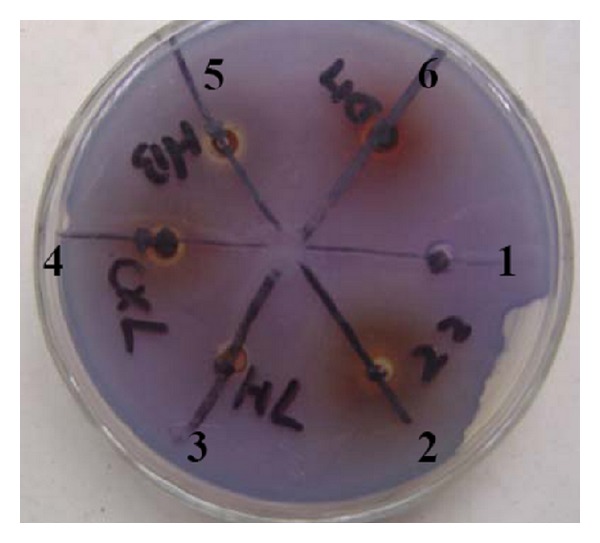
Antiquorum sensing activity of TCHMs water extract against *C. violaceum* CV12472. The extracts shown are (1) negative control; (2) *Fructus gardeniae*; (3) *Rhizoma coptidis*; (4) *Andrographis paniculata*; (5) *Cortex phellodendri Chinensis*; (6) *Rhubarb*.

Initial screening of TCHMs water extracts for anti-QS activities was done using the *C. violaceum *CV12472 as preliminary bioassay. *C. violaceum *CV12472 is able to produce the purple pigment violacein unless its QS signals were inhibited. Three out of five TCHMs displayed QS inhibitory activity as indicated in Figure 1. On the basis of this result, the extracts of *Fructus gardeniae, Andrographis paniculata,* and *Rhubarb* were used to study their QS inhibitory effect on *P. aeruginosa*.

### 3.2. Effect of TCHMs Extract on the Growth of *P. aeruginosa*

MICs of five TCHMs including three which have anti-QS activity were assessed using doubling dilution method with the concentrations varying from 0.0039 to 0.500 g/mL. The MICs of *Fructus gardeniae* and *Rhizoma coptidis* were more than 0.500 mg/mL, *Andrographis paniculata* and *Cortex phellodendri Chinensis* were 0.500 mg/mL and 0.250 mg/mL of *Rhubarb *(Table 1).

Based on the MIC, the sub-MIC concentrations of three TCHMs which have quorum sensing inhibitory activity were used to detect their effect on the growth of *P. aeruginosa* PAO1. The concentrations of *Rhubarb*, *Fructus gardenia,* and *Andrographis paniculata* were 0.0625 g/mL, 0.25 g/mL, and 0.125 g/mL, respectively. In general, the growth of PAO1 was not significantly inhibited by the three different TCHMs at concentration detected in LB medium.

### 3.3. Inhibition of QS-Dependent Virulence Determinants of PAO1

#### 3.3.1. Swimming Assay

**Figure 2:**
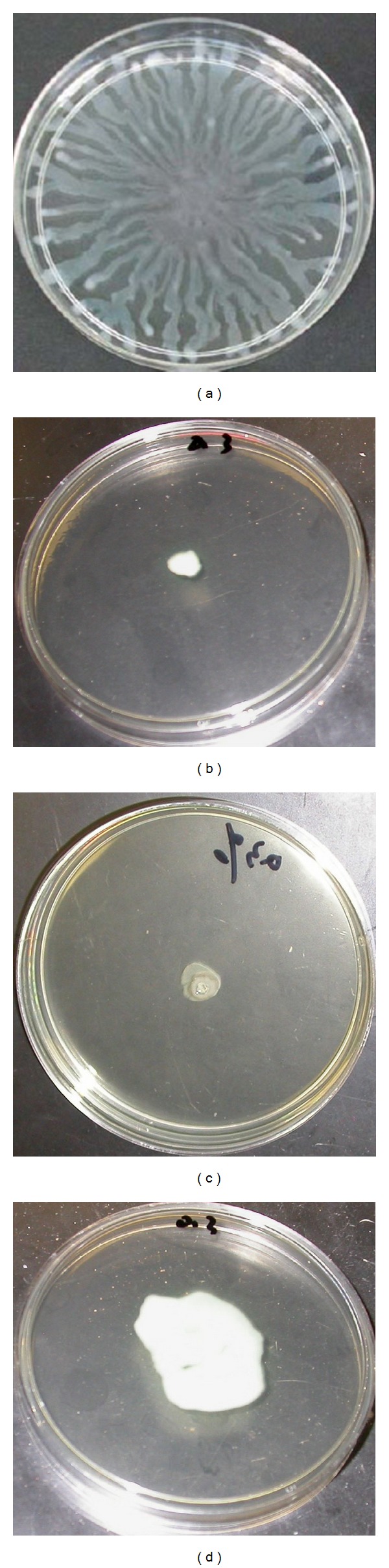
Inhibition of *P. aeruginosa* PAO1 swarming by the water extract of TCHMs. (a) *P. aeruginosa* PAO1 (no addition of the extracts); (b) *Fructus gardeniae* extract; (c) *Rhubarb* extract; (d) *Andrographis paniculata* extract.

Flagella motility-dependent swimming is also regulated by QS. A reduction in swimming area compared with control plate suggests presence of antiquorum sensing compounds. Three TCHMs which have antiquorum sensing activity and are selected distinctly reduced the swimming area in the reporter PAO1, suggesting that these TCHMs can inhibit the swimming motility of PAO1 (Figure 2).

#### 3.3.2. Total Proteolytic Activity and Elastase Activity

**Figure 3:**
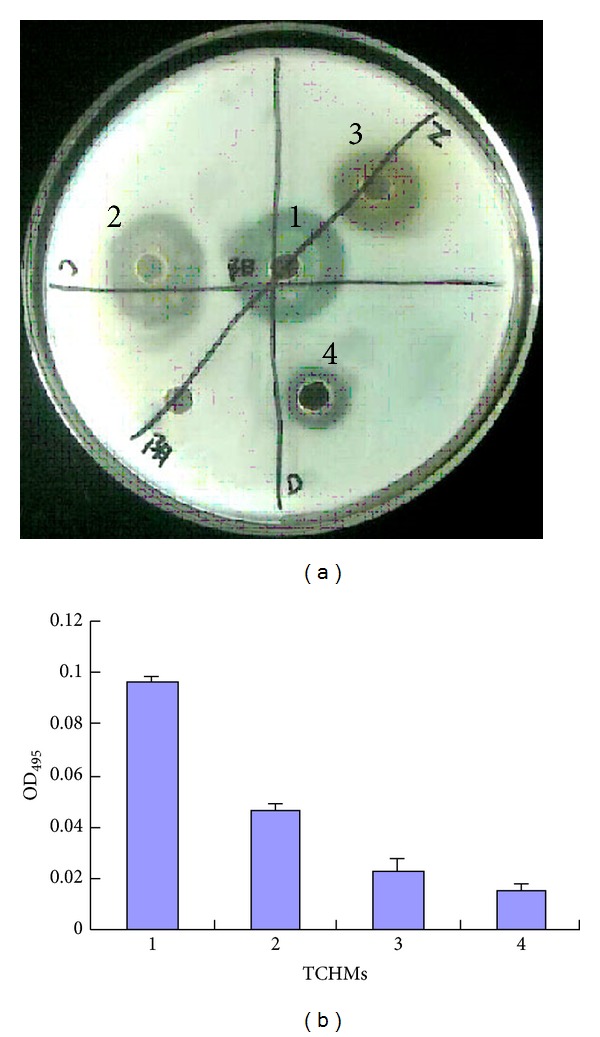
Effect of TCHMs on *P. aeruginosa* PAO1 protease activities. The total proteolytic activity on skim milk plate (a) and elastase activities were monitored in the absence and presence of three TCHMs (b). Mean values of triplicate independent experiments and SD are shown. (1) *P. aeruginosa* PAO1 (no addition of the extracts); (2) *Rhubarb* extract; (3) *Andrographis paniculata* extract; (4) *Fructus gardeniae* extract.

The ability of TCHMs in reducing QS-dependent protease and elastin-degrading elastase activity was assessed. As shown in Figure 3, a decrease in protease activity was observed in the supernatant of TCHMs-treated PAO1, with that of untreated PAO1 supernatant. The elastase activity was inhibited at a maximum of 84.38% when PAO1 was grown in the presence of *Fructus gardeniae* extract. However, other plant extracts of *Andrographis paniculata* and *Rhubarb* extract showed a moderate reduction of elastase activity up to 76.04% and 52.08%, respectively (Figure 3).

#### 3.3.3. Pyocyanin Assay

**Figure 4:**
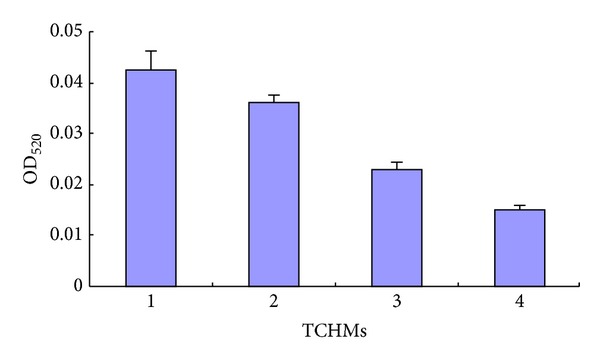
Effect of water extract TCHMs on *P. aeruginosa* PAO1 pyocyanin production. Data are represented as the percentage inhibition of biofilm formation. Mean values of triplicate independent experiments and SD are shown. (1) *P. aeruginosa* PAO1 (no addition of the extracts); (2) *Rhubarb* extract; (3) *Andrographis paniculata* extract; (4) *Fructus gardeniae* extract.

In order to analyze the efficiency of the TCHMs to reduce QS-dependent pyocyanin production, the PAO1 cells were cultivated in the presence and the absence of test TCHMs. A significant decrease in pyocyanin production of PAO1 was observed to the level of 35.21% after treatment with *Fructus gardeniae* extract. Extract of *Andrographis paniculata* and *Rhubarb* showed a reduction in pyocyanin production of PAO1 to 53.99% and 84.51%, respectively (Figure 4). 

#### 3.3.4. Biofilm Formation

**Figure 5:**
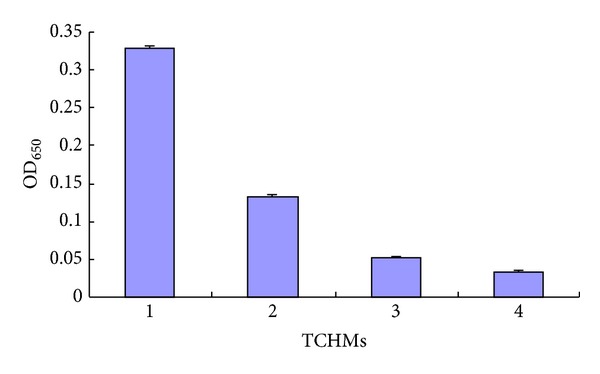
Effect of TCHMs on *P. aeruginosa* PAO1 biofilm formation as quantified by crystal violet staining and measuring at A650 nm. Mean values of triplicate independent experiments and SD are shown. (1) *P. aeruginosa* PAO1 (no addition of the extracts); (2) *Rhubarb* extract; (3) *Andrographis paniculata* extract; (4) *Fructus gardeniae* extract.

There was a significant decrease in biofilm formation when PAO1 was grown in the presence of *Fructus gardeniae* extract (89.63% decrease), *Andrographis paniculata* extract (84.15% decrease), and *Rhubarb* extract (59.76% decrease) when compared to the control (Figure 5). 

## 4. Discussion

The ability to control growth of pathogenic and otherwise unwanted bacteria was one of the greatest technical achievements of the last century. Antibiotics that aim at killing or inhibiting growth of microorganisms have been applied in animal and human health and particularly in the treatment of bacterial infections. This strategy has an obvious drawback: when growth of bacteria is blocked, the bacteria are under harsh selective pressure to develop resistance, so as the results, more and more drug-resistant bacteria come out, and the antibiotics lost their effects. The discovery and elucidation of bacterial QS systems have been paralleled by growing interest in the ability to manipulate signal reception and transduction. QS systems of pathogens are central regulators for the expression of virulence factors and represent highly attractive targets for the development of novel therapeutics. QSIs can competitively inhibit QS signaling system, providing an opportunity to develop new drugs against these targets to combat pathogens [19]. In *P. aeruginosa* PAO1, the *LasIR* and *RhlIR* systems coordinate the expression of various genes including production of pyocyanin pigment, virulence factors such as LasA staphylolytic protease, LasB elastase, and biofilm formation [11, 18, 20]. Recently, several potential QSIs have been discovered from various resources [21]. In the present study, it was intended to find out natural QS signal antagonists from the traditional Chinese herbal medicines.

Traditional Chinese herbal medicines have been used since ancient time in China to treat diseases; the type of “Qingre” herb made their effects by the mechanisms known as “cleansing heat”, “drying moisture”, and “removing toxins” [22]. They possess various medicinal effects including antiviral, antibacterial antitumor, sedative, antipyretic, antihypertensive, diuretic, and haemostatic effects. In this study, we found that three of the “Qingre” TCHMs also possessed anti-QS activity. The production of violacein pigment in *C. violaceum* CV12472, which is mediated by quorum sensing, was inhibited by the water extracts from *Fructus gardeniae*, *Andrographis paniculata,* and *Rhubarb* (Figure 1). These findings indicated the potential of TCHMs as a source of anti-QS compounds and highlight the importance of evaluating the unexplored diversity of TCHMs for such activity. Plant extracts showed QSI activity also reported by others. Pyrogallol extracted from medicinal plants such as *Emblica officinalis* and its analogues exhibit antagonism against AI-2 [23]. *Curcuma longa* produces curcumin, which inhibits the expression of virulence genes of *P. aeruginosa* PAO1 [24]. Extracts from different plant parts like leaves, flowers, fruit, and bark of *Combretum albiflorum*, *Laurus nobilis*, and *Sonchus oleraceus* were also found to possess anti-QS activities [25, 26]. 

Pathogenicity of *P. aeruginosa* involved various factors such as adhesins, including the type IV pili, and flagella, secreted extracellular virulent factors, such as pyocyanin and protease, which can cause substantial damage to host cells; and tissues; biofilm formation. QS-regulated swarming motility has been characterized as form of flagella-dependent movement on a viscous environments. *P. aeruginosa* require flagella for swarming motility which promote bacterial adhesion to different surfaces or host cells; it is the first step of biofilm formation of invasion. Our results showed that TCHMs extracts can inhibit swarming motility of *P. aeruginosa* PAO1 (Figure 2). Similar findings were reported by Koh and Tham [27] as they found that four of the traditional Chinese herbs inhibit the swarming motility of PAO1, *Areca catechu* (seed) extract reduced swarming the greatest (79% reduction compared with control), and *Panax notoginseng* (root) (flower) and *Prunus armeniaca* (kernel of seed) reduced swarming ability of PAO1 50%, 32%, 29% respectively.

The effect of TCHMs extracts against AHL-mediated total proteolytic activity of PAO1 revealed that all three selected medicines reduced the enzyme activity (Figure 3). A similar inhibitory effect on total protease activity in PAO1 was observed earlier with the extracts of *A. alopecuroidea *(up to 95%), *Arctostaphylos uva-ursi, *and *Senecio formosus* results which showed that the protease activity was significantly reduced by *A. comosus *extract (92.64%), moderately with *M. paradisiaca *and *M. zapota *(48.16 and 35.50%, resp.) and to a very little extent with *O. sanctum *(11.12%) [28, 29]. Adonizio et al. research showed that the QS inhibitory activity of *C. erectus, Tetrazygia bicolor, C. viminalis,* and *C. hypericifolia *on LasA protease production has recently been shown to reduce up to 94, 89, 71, and 49%, respectively [18]. 

It was also observed that other AHL-mediated phenomena in PAO1 such as formation of biofilm and pyocyanin production were also reduced by these extracts (Figures 4 and 5). 

Aqueous extracts of edible plants and fruits such as *Ananas comosus*, *Musa paradisiaca*, *Manilkara zapota,* and Ocimum sanctum proved to be QSI against violacein production by *C. violaceum* and pyocyanin pigment, staphylolytic protease, elastase production, and biofilm formation abilities of *P. aeruginosa* PAO1 [29].

In conclusion, we have demonstrated that traditional Chinese herbal medicines with “Qingre” characteristics have the ability to inhibit the QS activity. The QS inhibition property of these traditional Chinese herbal medicines could be used as an alternative to conventional antibiotic treatment and to evade the development of antibiotic resistance among bacterial pathogens.

## Figures and Tables

**Table 1:** MIC of water extracts TCHMs on *P. aeruginosa*.

TCHMs	Concentration (g/mL)								Negative control	Positive control
	0.500	0.250	0.125	0.0625	0.0313	0.0156	0.0078	0.0039		
(1)	+	+	+	+	+	+	+	+	−	+
(2)	+	+	+	+	+	+	+	+	−	+
(3)	−	+	+	+	+	+	+	+	−	+
(4)	−	+	+	+	+	+	+	+	−	+
(5)	−	−	+	+	+	+	+	+	−	+

(1) *Fructus gardeniae*; (2) *Rhizoma coptidis*; (3) *Andrographis  paniculata*; (4) *Cortex phellodendri *Chinensis; (5) *Rhubarb*.
